# Would New SARS-CoV-2 Variants Change the War against COVID-19?

**DOI:** 10.3390/epidemiologia3020018

**Published:** 2022-04-29

**Authors:** Elrashdy M. Redwan, Fatma Elrashdy, Alaa A. A. Aljabali, Wagner Baetas-da-Cruz, Debmalya Barh, Adam M. Brufsky, Sk. Sarif Hassan, Kenneth Lundstrom, Ángel Serrano-Aroca, Kazuo Takayama, Murtaza M. Tambuwala, Bruce D. Uhal, Vladimir N. Uversky

**Affiliations:** 1 Department of Biological Sciences, Faculty of Sciences, King Abdulaziz University, P.O. Box 80203, Jeddah 21589, Saudi Arabia; 2 Therapeutic and Protective Proteins Laboratory, Protein Research Department, Genetic Engineering and Biotechnology Research Institute, City of Scientific Research and Technological Applications, New Borg El-Arab, Alexandria 21934, Egypt; 3 Department of Endemic Medicine and Hepatogastroenterology, Cairo University, Cairo 12111, Egypt; fatmaelrashdy@kasralainy.edu.eg; 4 Department of Pharmaceutics and Pharmaceutical Technology, Faculty of Pharmacy, Yarmouk University, Irbid 566, Jordan; alaaj@yu.edu.jo; 5 Translational Laboratory in Molecular Physiology, Centre for Experimental Surgery, College of Medicine, Federal University of Rio de Janeiro (UFRJ), Rio de Janeiro 21941-901, RJ, Brazil; wagner.baetas@gmail.com; 6 Institute of Integrative Omics and Applied Biotechnology (IIOAB), Nonakuri, Purba Medinipur 721172, West Bengal, India; dr.barh@gmail.com; 7 Laboratório de Genética Celular e Molecular, Departamento de Genética, Ecologia e Evolução, Instituto de Ciências Biológicas, Universidade Federal de Minas Gerais, Belo Horizonte 31270-901, MG, Brazil; 8 UPMC Hillman Cancer Center, Department of Medicine, Division of Hematology/Oncology, University of Pittsburgh School of Medicine, Pittsburgh, PA 15232, USA; brufskyam@upmc.edu; 9 Department of Mathematics, Pingla Thana Mahavidyalaya, Maligram, Paschim Medinipur 721140, West Bengal, India; sarimif@gmail.com; 10 PanTherapeutics, Rte de Lavaux 49, CH 1095 Lutry, Switzerland; lundstromkenneth@gmail.com; 11 Biomaterials and Bioengineering Lab, Centro de Investigación Traslacional San Alberto Magno, Universidad Católica de Valencia San Vicente Mártir, 46001 Valencia, Spain; angel.serrano@ucv.es; 12 Center for iPS Cell Research and Application (CiRA), Kyoto University, Kyoto 606-8507, Japan; kazuo.takayama@cira.kyoto-u.ac.jp; 13 School of Pharmacy and Pharmaceutical Science, Ulster University, Coleraine BT52 1SA, UK; m.tambuwala@ulster.ac.uk; 14 Department of Physiology, Michigan State University, East Lansing, MI 48824, USA; bduhal@gmail.com; 15 Department of Molecular Medicine and USF Health Byrd Alzheimer’s Research Institute, Morsani College of Medicine, University of South Florida, Tampa, FL 33612, USA

**Keywords:** SARS-CoV-2, COVID-19, variant, sublineage, transmission, immunity, infection, vaccination, non-pharmaceutical interventions

## Abstract

The scientific, private, and industrial sectors use a wide variety of technological platforms available to achieve protection against SARS-CoV-2 (severe acute respiratory syndrome coronavirus 2), including vaccines. However, the virus evolves continually into new highly virulent variants, which might overcome the protection provided by vaccines and may re-expose the population to infections. Mass vaccinations should be continued in combination with more or less mandatory non-pharmaceutical interventions. Therefore, the key questions to be answered are: (i) How to identify the primary and secondary infections of SARS-CoV-2? (ii) Why are neutralizing antibodies not long-lasting in both cases of natural infections and post-vaccinations? (iii) Which are the factors responsible for this decay in neutralizing antibodies? (iv) What strategy could be adapted to develop long-term herd immunity? (v) Is the Spike protein the only vaccine target or is a vaccine cocktail better?

## 1. Can SARS-CoV-2 Infection Provide Lifelong Immunity?

Adaptive immune responses triggered by SARS-CoV-2 infections, including B and T lymphocyte cell response, can induce immunological memory during primary infection to prevent or decrease disease severity on repeated exposures to the same pathogen. On reinfection, antibodies produced by short-lived plasma cells derived from B lymphocytes bind to and block the recognized virus, and the long-lasting bone marrow plasma cells (BMPCs) become resident in the bone marrow and retain the ability to produce antibodies for decades [1].

The SARS-CoV-2 Immunity & REinfection EvaluatioN (SIREN) study enrolled 8278 SARS-CoV-2 seropositive and 17,383 seronegative healthcare workers in England between 18 June 2020, and 31 December 2020 [2]. The study demonstrated that individuals with a previous history of SARS-CoV-2 infection exhibited an 84% lower infection risk than the seronegative individuals, with a median protective effect lasting 7 months following primary infection [2].

However, this “long-lasting” immunity may not protect against reinfection, and despite huge numbers of infections and deaths worldwide caused by SARS-CoV-2, herd immunity could not be achieved in communities with the previous infection of most of its population. One factor which affects protection against reinfections is the severity of primary infections. In severe SARS-CoV-2 infection, the immune response may be impaired, which will decrease affinity maturation, memory quality, and quantity [3]. In contrast, in cases of mild COVID-19 (coronavirus disease 2019) disease and quick recovery, the efficient antibody immune response that cleared symptoms rapidly remains stable for a long time after infection [4]. Another factor is the decrease/waning in antibody levels with time, which was demonstrated in seven individuals five months after SARS-CoV-2 infection [5].

It has also been reported that circulating anti-spike IgG antibodies remain detectable one year after hospitalization of COVID-19 patients, and these higher antibody titers and disease severity were associated with increased durability of detectable antibodies [6]. Although the total circulating anti-SARS-CoV-2 antibodies wane over a few months, these antibodies could be detected up to one year after infection, even in patients with mild COVID-19 [7]. The levels of circulating neutralizing antibodies correlate with the duration and infection severity but not with patient age [8]. Many parameters such as but not limited to patient age, symptomatic or asymptomatic phenotype, symptomatic grade, duration of sampling, type of sample, and type of evaluation methodology used have not been broadly discussed in publications [9]. This has generated existing controversies regarding the durability of the acquired immunity.

Another concern about assessing long-lasting immunity after primary SARS-CoV-2 infection is the difficulty of identifying the precise incidence of SARS-CoV-2 reinfection [10,11]. Viral genome sequencing from primary and secondary infections is required to ensure that they are two separate events and repeated PCR (polymerase chain reaction) testing is required to confirm reinfection. Individuals who have tested positive in PCR assays should show a negative PCR result and then test positive after reinfection. Moreover, reinfections were difficult to track during the first wave of the COVID-19 pandemic due to the overloaded testing capacity [10,11].

New SARS-CoV-2 variants may be the most important explanation for the immunity loss due to the presence of mutations making them more transmissible, more efficient at avoiding the host immune system and evading immunity elicited by primary infection. In fact, throughout the COVID-19 pandemic, many SARS-CoV-2 variants have appeared. Some are variants of interest (VOI) that may be associated with reduced efficacy of available treatments and vaccinations, and predicted increase in transmissibility or disease severity (e.g., B.1.427 (Epsilon), B.1.525 (Eta), and B.1.617.1 (Kappa)), and some are variants of concern (VOC) that may cause increase in transmissibility, more severe disease (e.g., increased hospitalizations or deaths), significant reduction in neutralizing antibodies generated during previous infection or vaccination (e.g., Alpha (B.1.1.7), Beta (B.1.351), Delta (B.1.617.2 and its sub-lineages AY.1, AY.2, and AY.3), Gamma (P.1), and most recently Omicron (B1.1.529)). The seriousness of this issue can be illustrated by the case in Manaus, Brazil, where SARS-CoV-2 infected more than 70% of the population by October 2020, which should have provided herd immunity. However, a surge of reinfection occurred in late December 2020, and early January 2021; i.e., about 7 months after the first wave [12,13]. The most likely explanation for the decrease in protective antibody levels and the reinfection of the population was the appearance of new more transmissible SARS-CoV-2 variants such as the B.1.1.7 (Alpha), B.1.351 (Beta), B.1.617.2 (Delta), and AY.1, AY.2, and AY.3 sub-lineages of Delta [12,13]. Another example is given by the Omicron variant, which was demonstrated to systematically escape neutralization by the existing vaccines, convalescent serum, and most therapeutic monoclonal antibodies (mAbs) [14,15,16]. In general, the accumulating evidence suggests that the existing vaccines might have limited protection against novel SARS-CoV-2 variants, and SARS-CoV-2 infection might show limited cross-variant immunity (e.g., see [17,18,19,20,21,22,23,24,25,26]).

Novel mutations will appear as long as the virus continues to spread. This emphasizes the need for precautionary measures to reduce the risk of infection, such as mask-wearing, physical distancing, hand hygiene, and surface sanitation. Obviously, among the very important constituents of the successful strategies to control pandemics are the existing and future anti-SARS-CoV-2 vaccines.

## 2. Can COVID-19 Vaccines Alone Stop the Pandemic?

Available SARS-CoV-2 vaccines stimulate adaptive immune responses against different SARS-CoV-2 Spike (S) proteins and decrease both symptomatic and asymptomatic disease incidence with the development of immunological memory.

The prospective SIREN study among staff working in publicly-funded hospitals in England demonstrated that the BNT162b2 mRNA vaccine could prevent both symptomatic and asymptomatic infection caused by the B1.1.7 (Alpha) variant by 70% (95% CI 55–85) 21 days after the first dose and 85% (74–96) 7 days after the second dose [27]. For the moment, the estimated time of lasting protection from severe disease after vaccination is up to 8 months, even with the decay of neutralizing antibody titers after this period [28].

Despite the high efficacy of available COVID-19 vaccines, SARS-CoV-2 infections can still occur in vaccinated individuals due to the decline in neutralizing antibody titers 7–8 months post-vaccination. Furthermore, the emerging new SARS-CoV-2 variants are one of the most important sources of post-vaccination infections [29]. The reported efficacy against the B.1.351 (Beta) variant first identified in South Africa is 57% against moderate-to-severe COVID-19, and 89% against severe COVID-19 for the Ad26.COV2.S vaccine, and zero percent against mild-to-moderate COVID-19 for the ChAdOx1 nCoV-19 vaccine [30]. Two cases out of 417 who had received the second dose of the BNT162b2 or the mRNA-1273 vaccines developed breakthrough infection two weeks after vaccination despite evidence of vaccine efficacy. Breakthrough infections correspond to cases when individuals test positive for COVID-19 after they have been fully vaccinated against the disease [31]. Subsequent viral sequencing revealed the E484K, T95I, del142–144, and D614G mutations in SARS-CoV-2 responsible for those breakthrough infections [32].

Concerns about not acquiring post-vaccination herd immunity also include the uneven distribution of vaccines among countries and within individual countries, and the age limit of vaccination (although vaccination is now recommended for everyone ages 5 years and older by the CDC (U.S. Centers for Disease Control and Prevention)). These factors make it more difficult to reach the levels of the vaccinated population needed to achieve herd immunity [33]. A study reported sequencing of over 2000 samples from COVID-19 patients below the age of 19 years and found more than 250 VOCs, with over 70% of the VOCs in children below the age of 12, including 33 cases of B.1.1.7 (Alpha) and 119 of B.1.429/B.1.427 (Epsilon) variants [34].

Another thing to consider is the possibility of virus spread by fully vaccinated individuals without manifesting breakthrough disease. Data released by the CDC reported that vaccinated people infected by the SARS-CoV-2 Delta variant could carry viral loads similar to those of unvaccinated people (https://www.cdc.gov/coronavirus/2019-ncov/variants/delta-variant.html (accessed on 10 December 2021)). Currently, it is not recommended to test vaccinated people following exposure to infection. However, highly tested groups, such as professional sport teams, demonstrated that asymptomatic breakthrough infections among vaccinated people might be higher than reported.

A crucial question related to vaccine efficacy against current and emerging SARS-CoV-2 is whether we are looking in the wrong place. Most of the current vaccine development focuses on the S protein as a target. Unfortunately, the virus mutates faster than humans engineer vaccines, so it might be a good idea to diversify our efforts. Obviously, the inactivated whole virus vaccines can generate a broad repertoire of antibody responses and the SARS-CoV-2-based vaccine from Sinopharm was approved by the WHO (the World Health Organization) for emergency use in May 2021. The surprising observation made in several published reports is that the comparison of the inactivated whole virus vaccines with mRNA vaccines revealed that the mRNA-based vaccines were systematically more efficient and durable than the inactivated whole virus vaccines [35,36,37,38]. Additionally, other initiatives, such as the combination of S, nucleoprotein, and ORF3a sequences have elicited neutralizing antibodies and demonstrated strong CD8 T cell responses against SARS-CoV-2 in most immunized individuals (https://ir.gritstonebio.com/static-files/6a7c26ca-06a6-4295-bf76-83948a341397 (accessed on 10 December 2021)). These observations suggested that the pan-vaccine approach could potentially be a way to outsmart the virus.

## 3. Breakthrough Infections of SARS-CoV-2 Variants and Community Herd Immunity Build-Up

### 3.1. Natural Infections

Many recovered patients have been reinfected worldwide, despite being among the population that had acquired immunity against the virus. However, the best-known example of breakthrough of a population with expected established herd immunity was observed in Manaus city (the capital of Amazonas state in northern Brazil), where from June 2020 to October 2020 the SARS-CoV-2 prevalence increased from 60% to more than 70%, a condition which may mirror achieving herd immunity [12]. By January 2021, Manaus had a huge resurgence in cases due to the emergence of a new variant known as P.1 (Gamma), which was responsible for nearly 100% of the new cases [13]. Although the population may have reached a high herd immunity threshold, there is still a risk of resurgence of new variants escaping protection. Sabino et al. attributed this to the waning of protective antibodies levels and emergence of new SARS-CoV-2 variants [12,13].

### 3.2. Vaccination

On a personal level, recovered patients can be reinfected, and the vaccinated persons can contract new infections (despite being previously infected or not). For example, the CDC reported that Massachusetts (USA) has already fully vaccinated more than 4 million people (out of 7.03 million total) (56.899%). The total rate of fully vaccinated people in the Barnstable County is 76% [31], with the vaccination levels by age category being as follows: 86% (75+), 77% (50–64), 80% (30–49), 62% (20–29), 64% (16–19), and 43% (12–15) (https://www.wcvb.com/article/massachusetts-covid-breakthrough-cases-delta-variant-pandemic-vaccine-data-charts-maps/37089843 (accessed on 10 December 2021)). However, the Department of Public Health of Massachusetts reported 7737 total COVID-19 breakthrough cases in fully vaccinated individuals as of 3 August 2021. Among the breakthrough cases, 395 patients have been hospitalized, and 100 have died. Genomic sequencing of specimens from 133 patients revealed that this breakthrough was caused by the newly identified and highly transmissible SARS-CoV-2 variants. The B.1.617.2 (Delta) variant was identified in 119 (89%) patients, and one person was infected by the Delta AY.3 sublineage (1%). Since the youngest age group (12–15) showed the lowest coverage by one and/or two vaccine doses (43% vs. >60% in other age groups), these data indicated the need for higher vaccination levels in this group. Although many factors, such as hesitancy and shortage of vaccine supply (especially in the developing countries) [39] define low vaccination rates, vaccination is still the most important strategy to prevent severe illness, hospitalization, and death.

The emergence of breakthrough infections has urged many developed countries to call their citizens for additional vaccine booster doses (specifically for those older than 60 years of age) from the same or newly approved vaccines (combined vaccines). However, it is not clear whether such boosting will help overcoming the viral infectivity and/or transmissibility, especially related to emerging new variants. The COVID-19 pandemic incidences worldwide clearly indicate that it might not. Overall, it seems that this virus can overcome herd immunity (formed naturally and/or via vaccination), suggesting that it can prevail in the general population for a long time. Of note, young individuals may represent a live source for new infections, as most of them, when infected, are asymptomatic or show light to moderate symptoms.

As the WHO has emphasized, 90–99% of individuals infected with the SARS-CoV-2 virus develop detectable neutralizing antibodies within four weeks after infection (https://apps.who.int/iris/handle/10665/341241 (accessed on 28 April 2022)), the levels of which decline during 6–12 months post-infection. Of note, the acquired herd immunity would not protect from reinfection but would significantly enhance the chance of building up the hybrid immunity post vaccination with any type of vaccine [40]. In parallel, according to classical immunology, most vaccines can elicit durable immune responses. However, the open questions are: Why does herd immunity (from natural infection or vaccination) against SARS-CoV-2 decrease within 6–12 months? What are the mechanisms behind such a decay? Are they due to the viral factors, or host immunity factors, or vaccine ingredients or because of all these factors together?

The SARS-CoV-2-specific antibody titer kinetics and decay have been evaluated in individuals vaccinated with the BNT162b2 mRNA vaccine and in convalescent COVID-19 patients [41,42]. Although higher SARS-CoV-2 IgG antibody titers were observed in vaccinated individuals, their titers decreased by 38% each subsequent month. In contrast, less than 5% reduction was observed in convalescent patients. At 6 months post-vaccination, 16.1% of vaccinated individual exhibited antibody levels below the seropositivity threshold. In contrast, 9 months after testing positive for COVID-19, 10.8% of convalescent patients were below the <50 AU/mL threshold [41]. This suggested that different kinetics of antibody levels were detected between vaccinated individuals and convalescent patients, where the BNT162b2 mRNA vaccine provided higher initial antibody levels but faster exponential decrease. In another study, antibody titers demonstrated a 70% decrease in healthcare professionals 6 months post-vaccination, although the titers were one order of magnitude higher compared to seropositive individuals [42].

**Figure 1:**
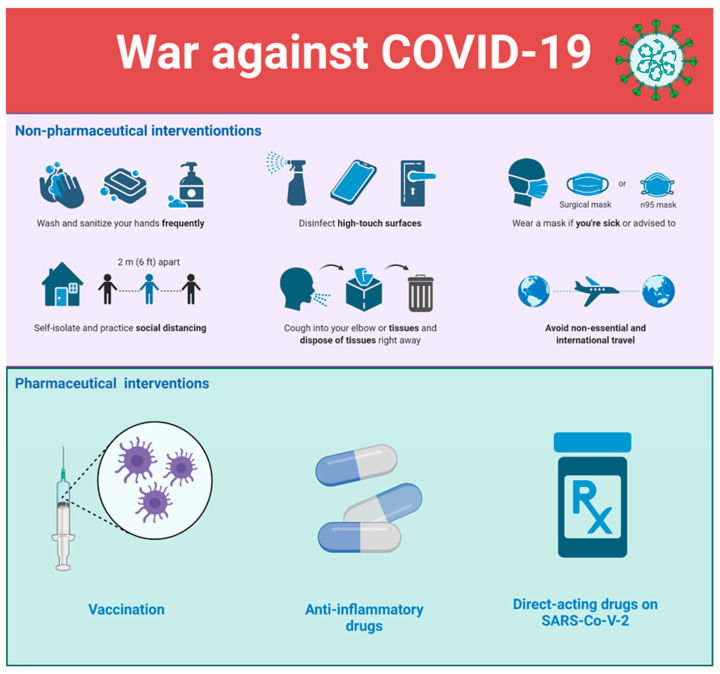
The war against COVID-19 includes multiple strategies. Both pharmaceutical and non-pharmaceutical interventions are needed now and may be needed for a long time.

Taking all these facts into account, it is clear that the war against COVID-19 should be conducted at multiple levels (see Figure 1), and our efforts to end virus transmission, complications, lockdowns, and disruption of the world economy should not be limited to only endorsing various approaches of vaccination (e.g., promoting different vaccines, encouraging booster doses, recommending combined vaccines, re-engineering existing vaccines to target emerging variants, etc.). We should also definitely use various antibody-based immunotherapeutic approaches, such as convalescent plasma and intravenous immunoglobulins to manage COVID-19 patients [43]. Moreover, one should not neglect the important roles of monoclonal antibody therapeutics (mAb), although they are expensive and not applicable for large populations. They are the key antiviral reagents for individuals refusing vaccination, immune compromised patients, or non-hospitalized patients with laboratory-confirmed SARS-CoV-2 infection showing mild to moderate COVID-19 and who are at high risk for progressing to severe disease and/or hospitalization [44,45,46,47,48]. Moreover, mAbs might be useful in cases where vaccine efficacy against new variants is insufficient. In fact, several anti-SARS-CoV-2 mAb combinations of bamlanivimab and etesevimab, casirivimab and imdevimab (REGEN-COV), sotrovimab, and a long-acting anti-SARS-CoV-2 mAb combination, tixagevimab and cilgavimab (Evusheld), have received Emergency Use Authorizations (EUAs) from the Food and Drug Administration (FDA). However, it has been shown that bamlanivimab and etesevimab and REGEN-COV have demonstrated reduced efficiency against the B.1.1.529 (Omicron) variant of SARS-CoV-2. Furthermore, although the efficacy against the original Omicron variant was retained in the case of sotrovimab [49,50], the appearance of the Omicron BA.2 variant has changed this situation, and FDA restricted the use of sotrovimab, as the authorized dose of this mAb is unlikely to be effective against the BA.2 sub-variant [51]. All this indicates that the battle with COVID-19 is challenging, and victory requires utilization of all possible means.

In light of these considerations, we also need to place greater emphasis on the various hygienic precautions. Therefore, non-pharmaceutical interventions and protective precautionary actions such as use of protective masks, frequent disinfection of public areas, and social distancing, especially indoors, should continue to remain an important and mandatory part of our daily life. One should keep in mind that when the non-pharmaceutical interventions are relaxed when the majority of the population has already been vaccinated, the probability of the emergence of a new resistant strain is greatly increased. Therefore, individuals should be encouraged to maintain the non-pharmaceutical interventions and transmission-reducing behaviors throughout the entire vaccination period [52]. Obviously, the development of novel drugs directly targeting the SARS-CoV-2 would significantly help reducing viral transmission. Furthermore, we need to understand better what represents a protective or fully protective immunity threshold for SARS-CoV-2 infection, and, similar to almost all approved vaccines for human beings, the world needs to achieve a consensus on the protective immunity threshold [53]. To this end, standard or consensus methods that would consider various correlations of different in vitro and in vivo data are needed to estimate the quality and quantity of protective immunity against SARS-CoV-2 [9,54,55].

## Data Availability

Not applicable.
